# Low-Temperature Properties of the Magnetic Sensor with Amorphous Wire

**DOI:** 10.3390/s20236986

**Published:** 2020-12-07

**Authors:** Dongfeng He, Kensei Umemori, Ryuichi Ueki, Takeshi Dohmae, Takafumi Okada, Minoru Tachiki, Shuuichi Ooi, Makoto Watanabe

**Affiliations:** 1 National Institute for Materials Science, 1-2-1 Sengen, Tsukuba, Ibaraki 305-0047, Japan; TACHIKI.Minoru@nims.go.jp (M.T.); OOI.Shuuichi@nims.go.jp (S.O.); WATANABE.Makoto@nims.go.jp (M.W.); 2 High Energy Accelerator Research Organization (KEK), 1-1 Oho, Tsukuba, Ibaraki 305-0801, Japan; kensei.umemori@kek.jp (K.U.); ryuichi.ueki@kek.jp (R.U.); dohmae@post.kek.jp (T.D.); okadat@post.kek.jp (T.O.); 3 School of High Energy Accelerator Science, The Graduate University for Advanced Studies (SOKENDAI), Shonan Village, Hayama, Kanagawa 240-0193, Japan

**Keywords:** magnetic sensor, amorphous wire, FeCoSiB, liquid helium

## Abstract

We found that a magnetic sensor made of a coil wound around a 5 ϕ0.1 mm (Fe_0.06_Co_0.94_)_72.5_Si_2.5_B_15_ (FeCoSiB) amorphous wire could operate in a wide temperature range from room temperature to liquid helium temperature (4.2 K). The low-temperature sensing element of the sensor was connected to the room-temperature driving circuit by only one coaxial cable with a diameter of 1 mm. The one-cable design of the magnetic sensor reduced the heat transferring through the cable to the liquid helium. To develop a magnetic sensing system capable of operating at liquid helium temperature, we evaluated the low-temperature properties of the FeCoSiB magnetic sensor.

## 1. Introduction

Some low-temperature experiments, such as flux expulsion experiments of superconductors [1,2], not only require magnetic sensors capable of operating at liquid helium temperature (4.2 K), but also require a sensor with a high dynamic range of several tens of Gauss, a high magnetic field resolution of more than 0.1 milligauss (mG), and good linearity. Superconducting quantum interference devices (SQUIDs) [3,4] are often used to measure weak magnetic fields at low temperature. However, the high price, complex operation, easy damage and small dynamic range limit the applications of SQUIDs in some fields. Jakub Jankowski et al. [5] developed a hall sensor using a heavily n-doped InSb layer epitaxially grown on GaAs. The working temperature range of the sensor extended from −270 to +300 °C. However, the magnetic field sensitivity was about 1000 times worse than that of high-sensitivity fluxgate magnetic sensors, magneto resistance (MR) magnetic sensors or magneto impedance (MI) magnetic sensor.

Due to their high sensitivity, fluxgate, MR and MI magnetic sensors have been used for the applications of bio-magnetic sensing [6,7], communication [8], nondestructive evaluation [9,10] and automotive [11]. Some fluxgate [12,13] and anisotropic magneto resistance (AMR) sensors [14,15] could even operate at 4.2 K. To develop multi-channel application systems, the number of the connection wires should be as few as possible, but four wires are needed for the fluxgate sensor and eight wires are needed for the AMR sensor to connect the sensing elements to the driving circuits. High-sensitivity magnetic sensors have been developed using amorphous wire made of the ferromagnetic material of (Fe_0.06_Co_0.94_)_72.5_Si_2.5_B_15_ (FeCoSiB) [16,17,18,19]. We previously reported the high-sensitivity FeCoSiB amorphous wire magnetic sensor. This magnetic sensor was composed of a FeCoSiB amorphous wire and a coil wound around it. A DC current and an AC current were used to bias the sensor through the coil. When the amorphous wire was near the saturation point by adjusting the DC bias current, the amplitude of the AC signal changed via the applied magnetic field. About 20 pT/√Hz magnetic field resolution at white noise frequency was achieved in the beginning [20]; then, it was improved to 6 pT/√Hz by using a resonant circuit before the preamplifier [21], and the dynamic range of ±10 Gauss was obtained by using a feedback method [22]. We tested the magnetic sensor at liquid nitrogen temperature and liquid helium temperature, and found that our FeCoSiB magnetic sensor could operate well in a wide temperature range from room temperature to 4.2 K. Table 1 shows the comparison of the magnetic sensors with wide operating temperature ranges: the fluxgate sensor [23,24], the AMR sensor [25,26] and the amorphous wire magnetic sensor (NIMS sensor) [21].

Due to the small size and high sensitivity of the NIMS magnetic sensor, it is possible to use this sensor for bio-magnetic measurement, nondestructive evaluation [27], automotive applications, and security check. Another advantage of the NIMS FeCoSiB amorphous wire magnetic sensor is that only one coaxial cable is used to connect the sensing element and the driving circuit. This one-cable design could effectively reduce the heat transferring through the cable to the liquid helium; thus, it would be suitable for constructing a multi-channel magnetic mapping system. To develop a high-sensitivity magnetic sensing system capable of operating at liquid helium temperature, we evaluated the low-temperature properties of the magnetic sensor with the FeCoSiB amorphous wire.

## 2. Methods

### 2.1. Measuring the Magnetic Hysteresis Loop (B–H Characteristics) of the FeCoSiB Amorphous Wire

**Figure 1:**
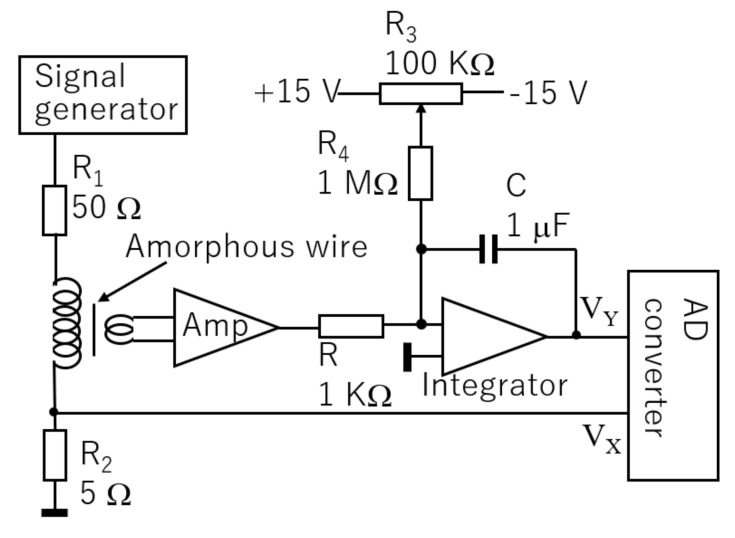
Schematic block diagram of the circuit for measuring the magnetization characteristics of the FeCoSiB amorphous wire.

Due to the small diameter of 0.1 mm and the small length of the FeCoSiB amorphous wire used for the sensor of 5 mm, it is difficult to measure the B–H characteristics using normal magnetization measurement equipment. A simple B–H characteristics measurement system was developed by the authors. Figure 1 shows the circuit diagram of the magnetization characteristic measurement for the amorphous wire.

We wound the coils using the ϕ0.1 mm copper wire. The magnetization coil was 80 turns with a length of 1 cm, and it was wound outside of a small plastic tube with an outer diameter of 0.5 mm and an inner diameter of about 0.3 mm. The FeCoSiB amorphous wire was placed inside the plastic tube. The length the of FeCoSiB amorphous wire was about 5 mm, which was smaller than the length of the magnetization coil; thus, the magnetic field applied to the amorphous wire was nearly uniform. The pickup coil was wound outside of the magnetization coil with 10 turns and a length of 1.5 mm. To estimate the flux easily from the voltage of the pickup coil, the length of the pickup coil was made smaller than the length of the FeCoSiB amorphous wire.

A 30 Hz sine wave signal produced by the signal generator was sent to the magnetization coil though a 50 Ω resistor. *V_X_* was used to measure the magnetization current and the magnetic field strength H. The signal from the pickup coil was amplified by 100, then sent to an integrator. The resistor *R* of 1 KΩ and the capacitor C of 1 μF were chosen in our experiments. *R*_3_ and *R*_4_ were used to compensate the drift and the offset of the amplifier. The output signal *V_Y_* of the integrator was used to measure the flux density B in the amorphous wire. The signals of *V_X_* and *V_Y_* were sent to an AD converter connected with a computer to plot the B–H characteristics. To obtain the values of B and H from the values of *V_X_* and *V_Y_*, some simple calculations must be performed.

The magnetic field strength H can be estimated by the following equation:

(1) $$H=\frac{N_{1}}{L}\cdot \frac{V_{X}}{R_{2}}$$

where *N*_1_ is the turn number of the magnetization coil; *L* is its length; *V_X_*/*R*_2_ is the magnetization current. In our experiments, *N*_1_ is 80, *L* is 10 mm, and *R*_2_ is 5 Ω.

The flux density *B* in the sample of the FeCoSiB amorphous wire can be estimated by the following equation:

(2) $$B=\frac{-V_{Y}\left(CR\right)}{GN_{2}}\cdot \frac{1}{\frac{\pi d^{2}}{4}}$$

where *N*_2_ is the turn number of the pickup coil; *G* is the gain of the amplifier; d is the diameter of the amorphous wire; *πd*^2^/4 is the cross area of the amorphous wire. In our experiments, *G* is 100, *N*_2_ is 10, *R* is 1 KΩ, *C* is 1 μF, and *d* is 0.1 mm.

### 2.2. Magnetic Sensor with FeCoSiB Amorphous Wire

**Figure 2:**
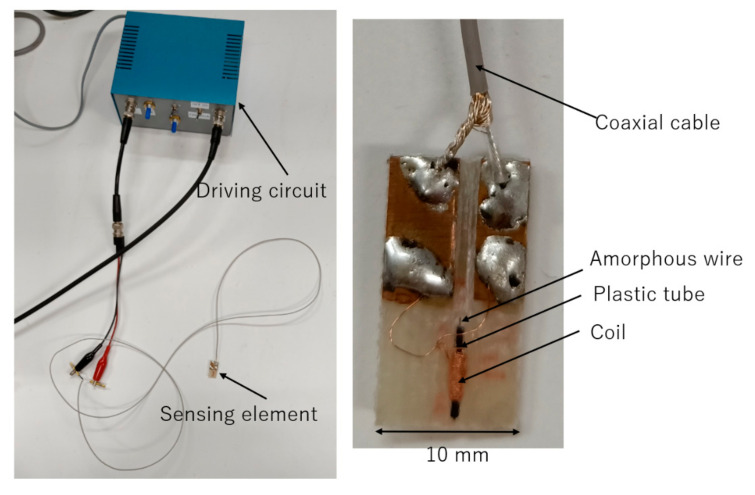
Magnetic sensor with FeCoSiB amorphous wire. The sensing element was connected to the driving circuit by only one coaxial cable.

**Figure 3:**
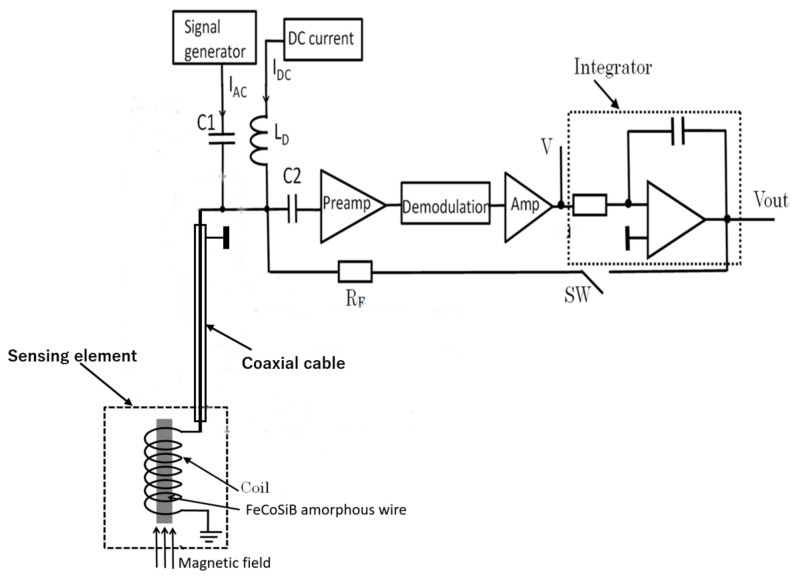
Feedback schematic block diagram of the magnetic sensor.

Figure 2 shows the sensing element and the driving circuit of the FeCoSiB magnetic sensor. For low-temperature application, the sensing element was placed in the liquid helium or liquid nitrogen; the driving circuit was at room temperature. As shown in Figure 2 and Figure 3, the sensing element of the magnetic sensor was connected to the driving circuit by only one coaxial cable with a diameter of about 1 mm.

The FeCoSiB amorphous wire had no electrical connection, and the cable was only soldered with the coil. Therefore, the sensor was very rigid, and there were no issues regarding connection break. The signal, the DC bias current, the AC bias current and the feedback current were all transmitted by this cable. They had different frequencies, different output resistances, or different input resistance; thus, these signals could be isolated by using some capacitors, inductors and the resistors. This one-cable design had the advantage of application at liquid helium temperature. It occupied a small space and had small heat transfer through the cable. The inductance of the coil was about L = 2 μH; therefore, the impedance of the coil was about 2πfL ≈ 12.6 Ω at 1 MHz. The length of the coaxial cable was 150 cm, and the resistance of the cable was about 0.7 Ω, which was much smaller than 12.6 Ω; thus, the cable had less influence on the signal amplitude of the FeCoSiB magnetic sensor.

Figure 3 shows the feedback schematic block diagram of the magnetic sensor. The applied magnetic field was automatically compensated by the magnetic field produced by the feedback current; thus, the operation of the magnetic sensor was locked to a fixed point. The dynamic range and the linearity of the sensor were improved by using feedback method [15]. The dynamic range of the magnetic sensor could be adjusted easily by changing the feedback resistance R_F_.

**Figure 4:**
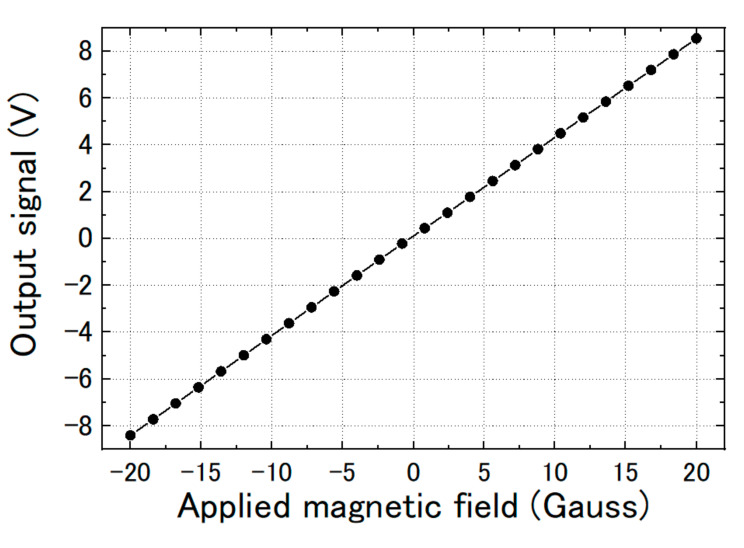
Linear response of the output signal to the applied magnetic field when the magnetic sensor had feedback.

Figure 4 shows the linear response of the output signal to the applied magnetic field when the magnetic sensor had feedback. The dynamic range was about ±20 Gauss. The transfer coefficient of voltage/magnetic field was about 420 mV/Gauss.

### 2.3. Experiments at Liquid Helium Temperature

**Figure 5:**
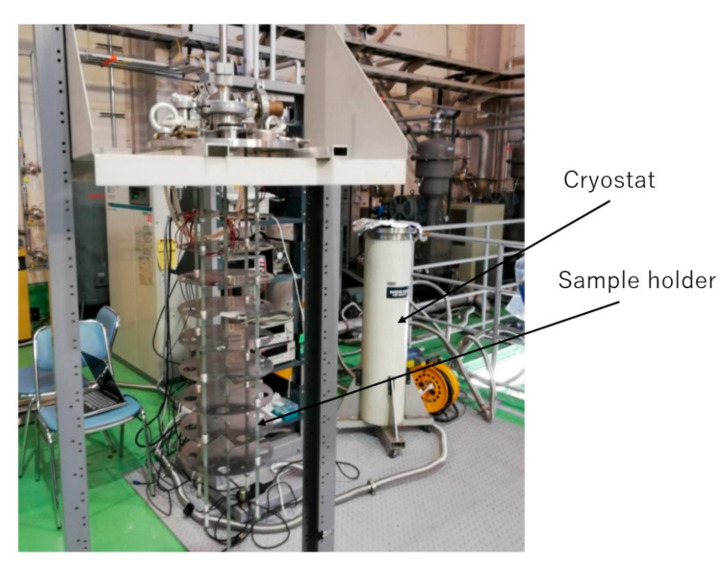
Cryostat and the sample holder for the low-temperature experiments.

A liquid helium cryostat was used for the test of the magnetic sensor at 4.2 K, as shown in Figure 5. The sensing element of the magnetic sensor was attached to the sample holder and was put into liquid helium for cooling. The driving circuit was placed outside the cryostat at room temperature. A thin coaxial cable with a diameter of about 1 mm is used to connect the sensing element of the magnetic sensor with the driving circuit.

A Helmholtz coil was used to apply a magnetic field to the magnetic sensor. As a comparison, a fluxgate magnetic sensor was placed near to the magnetic sensor to conduct the calibration. A group in the High Energy Accelerator Research Organization (KEK) aim to develop a magnetic field mapping system using low-temperature magnetic sensors to monitor the magnetic flux trapping and expulsion around the cavity during its cool-down and warm-up process. They tested the fluxgate and AMR sensors, but fluxgate sensor had a bigger size, and the AMR sensor needed 8 connection wires (Table 1); thus, the fluxgate and AMR sensors were not suitable to construct a multi-channel magnetic mapping system. To fulfill their requirements, the dynamic range of the magnetic sensor should be over 10 Gauss, the absolute accuracy should be greater than 100 nT, and the relative sensitivity should be greater than 10 nT. The NIMS amorphous wire magnetic sensor had only one cable connection, making its use possible for this application.

## 3. Results

### 3.1. Magnetiztion Characteristics of the FeCoSiB Amorphous Wire

**Figure 6:**
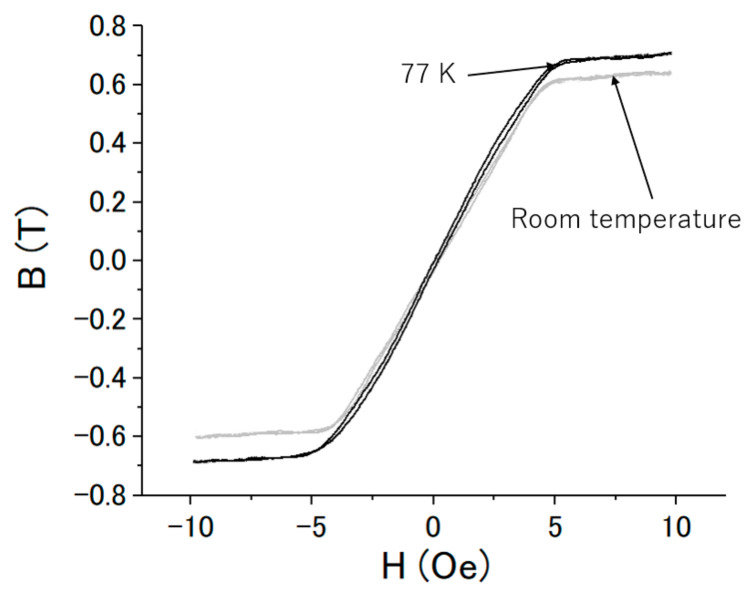
Magnetization characteristics of the FeCoSiB amorphous wire at liquid nitrogen temperature (77 K) and room temperature. The black line represents the result at 77 K, and the gray line represents the result at room temperature.

It is difficult to measure the B–H characteristics of the FeCoSiB amorphous wire at liquid helium temperature; thus, we only measured the B–H characteristics at room temperature and liquid nitrogen temperature (77 K). A low frequency current of 30 Hz was applied. The signals of *V_X_* and *V_Y_* in Figure 1 were measured. Using Equations (1) and (2), we calculated the flux density B, the magnetic field strength H and the magnetic flux density B from *V_X_* and *V_Y_*. Figure 6 shows the magnetization characteristics of the amorphous wire at room temperature and liquid nitrogen temperature. The changes in the B–H characteristics were small when the FeCoSiB amorphous wire was placed in liquid nitrogen. The magnetic field strength saturating the FeCoSiB amorphous wire was near 5 Oersted. The relative permeability was somewhat different at 77 K, and the saturation flux density increased from 0.6 T at room temperature to about 0.7 T at 77 K. The B–H curve at 4.2 K was not measured, but we tested the operation of the magnetic sensor at 4.2 K, and it operated well at 4.2 K.

### 3.2. Signal Amplitudes at Room Temperature and Liquid Nitrogen Temperature

**Figure 7:**
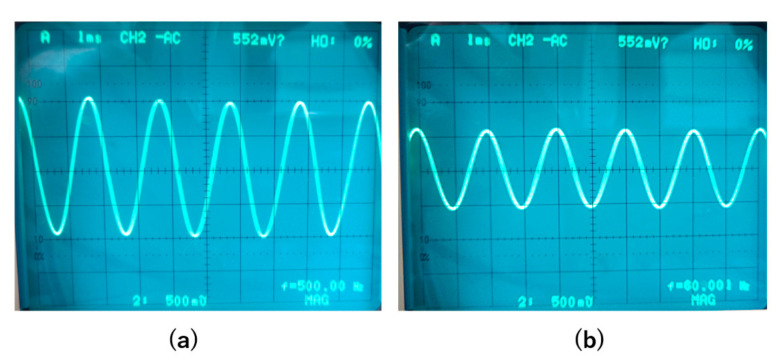
Output signals when the feedback was OFF. The frequency of the applied magnetic field was 500 Hz with a peak-to-peak amplitude of 0.5 Gauss. (**a**). Signal at room temperature. (**b**). Signal at liquid nitrogen temperature.

First, we tuned off the feedback of the magnetic sensor and measured the change in the output signal when changing the operation temperature from room temperature to liquid nitrogen temperature. Figure 7 shows the output signals when a 500 Hz magnetic field with a peak-to-peak amplitude of about 0.5 Gauss was applied. The signal amplitude was reduced by about half, from 2 to 1 V, when the sensor was moved from room temperature to 77 K.

**Figure 8:**
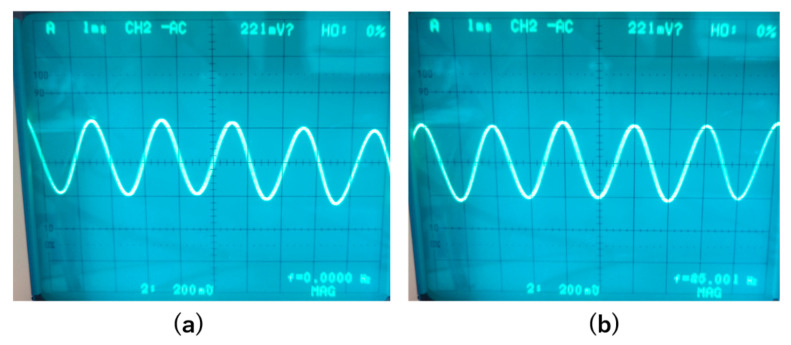
Output signal when the feedback was ON. The applied magnetic field was 500 Hz with an amplitude of 1 Gauss. (**a**). Signal at room temperature. (**b**). Signal at liquid nitrogen temperature.

Then, we turned on the feedback and also measured the output signals of the magnetic sensor at room temperature and at 77 K. The frequency of the applied magnetic field was also 500 Hz, and the amplitude was 1 Gauss. Figure 8 shows the results. With the feedback, the signal amplitude was mainly determined by the feedback resistance R_F_ and had less relation with gain of the amplifier; thus, the output signal had less change when changing the operation temperature from room temperature to liquid nitrogen temperature.

**Figure 9:**
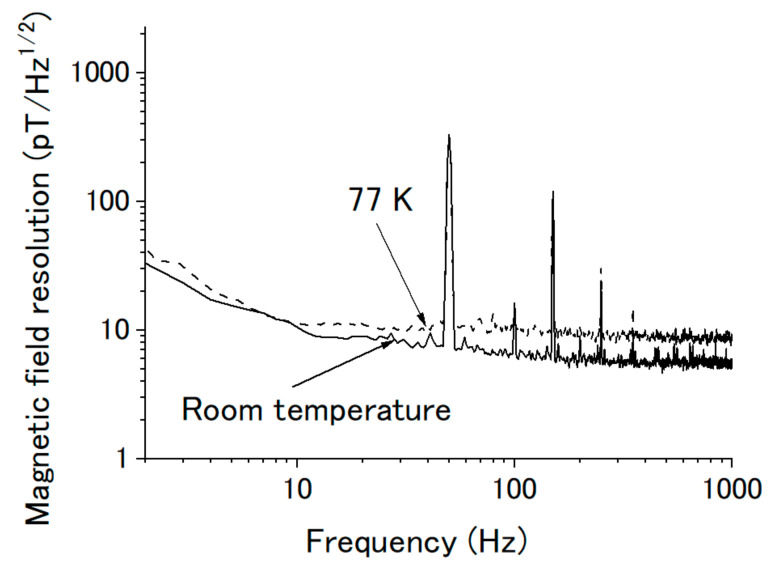
Magnetic field resolution of the magnetic sensor at room temperature (**solid line**) and 77 K (**dash line**).

We also measured the magnetic field resolution at room temperature and at 77 K. Figure 9 shows the results. The magnetic field resolution became worse when the magnetic sensor was at 77 K, from 6 to about 10 pT/√hz at the white frequency above 100 Hz, which was caused by the reduction of the signal at 77 K (Figure 7). However, the magnetic field resolution had almost no change at low frequency.

### 3.3. Magnetic Response of the FeCoSiB Magnetic Sensor

**Figure 10:**
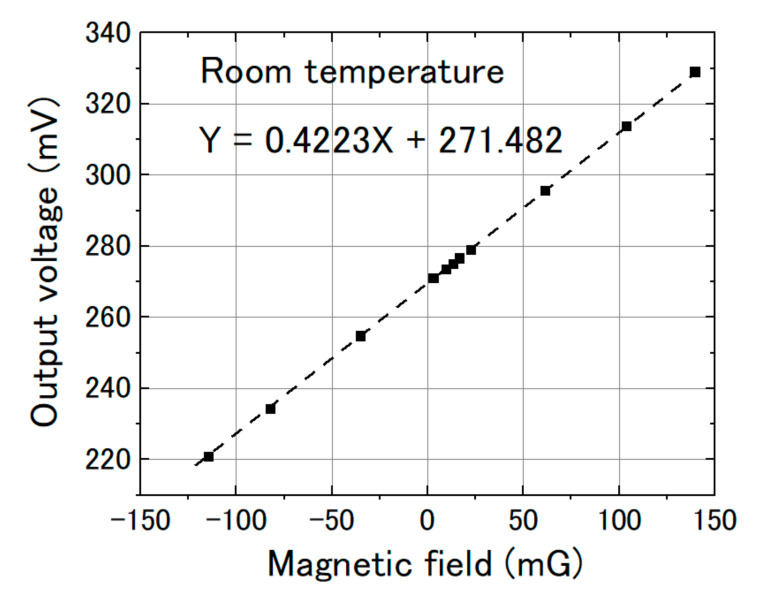
Sensor output vs. magnetic field at room temperature.

**Figure 11:**
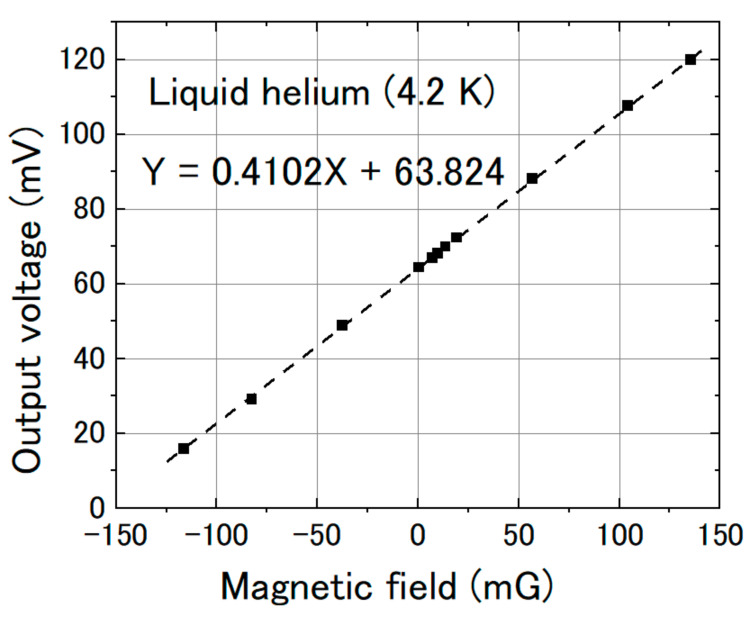
Sensor output vs. magnetic field at liquid helium temperature (4.2 K).

**Figure 12:**
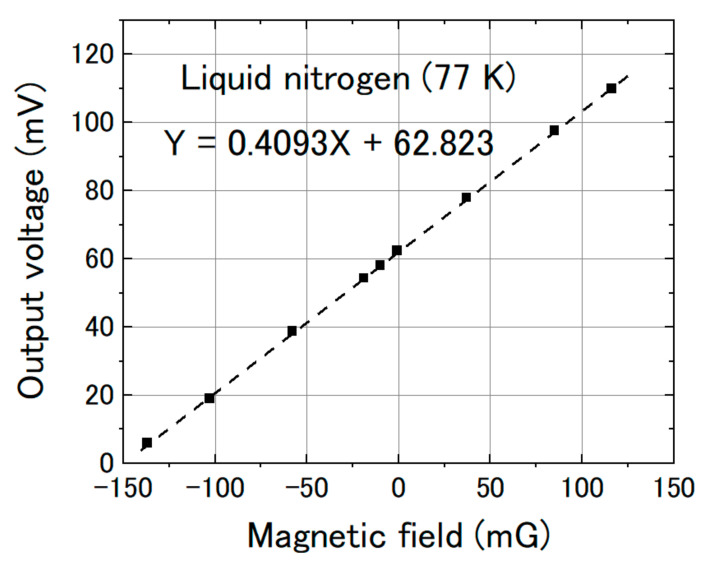
Sensor output vs. magnetic field at liquid nitrogen temperature (77 K).

We measured the magnetic response of the FeCoSiB magnetic sensor. The sensor was operated with feedback mode. The sensor was attached to the sample holder and placed into the cryostat. A cylinder type magnetic shielding was used to reduce the influence of the environmental noise. For the application in KEK, a DC magnetic field was measured; therefore, it needed high stability of the magnetic sensor for long-term measurement. The DC magnetic field was applied by a Helmholtz coil. In the beginning, we measured the magnetic response of the magnetic sensor at room temperature. Figure 10 shows the results. Then, we filled the cryostat with liquid helium and measured the magnetic response of the magnetic sensor at liquid helium temperature. Figure 11 shows the results at 4.2 K. As a comparison, we also measured the magnetic response at liquid nitrogen temperature (77 K). Figure 12 shows the results. The linearities of the responses were good at room temperature, 77 K and 4.2 K.

From Figure 10, Figure 11 and Figure 12, we can observe that the change in the slope of the magnetic response was small: from 0.4223 to 0.4092 and 0.4102 mV/mG when cooling the magnetic sensor from room temperature to 77 and 4.2 K. However, there was a big offset change when cooling the FeCoSiB magnetic sensor from room temperature to 77 and 4.2 K. The offset changed from 271.482 mV to 62.823 mV/mG and 63.824 mV. The driving circuit was kept at room temperature, and the offset produced by the driving circuit was not so big; therefore, the offset should come from the sensing element of the magnetic sensor. We provide an explanation for this in the Discussion Section.

**Figure 13:**
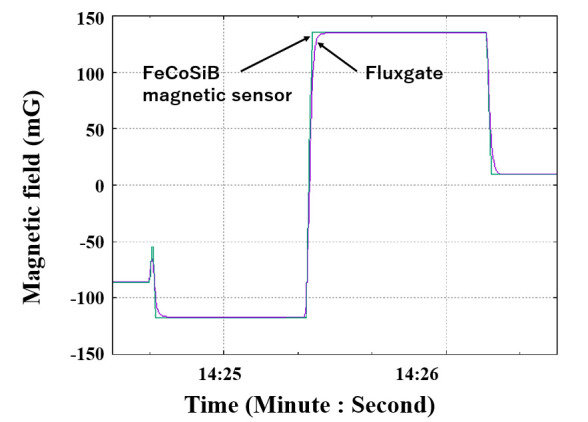
Outputs of the FeCoSiB magnetic sensor (**gray line**) and the fluxgate (**blue line**) for a rapid change in the magnetic field.

Figure 13 shows the calibrated outputs of the FeCoSiB magnetic sensor and the fluxgate magnetic sensor for a rapid change in the magnetic field. The gray line represents the output of the FeCoSiB magnetic sensor, and the blue line represents the output of the fluxgate magnetic sensor. They had very good agreement, and the FeCoSiB magnetic sensor had a faster response than the fluxgate magnetic sensor.

## 4. Discussion

**Figure 14:**
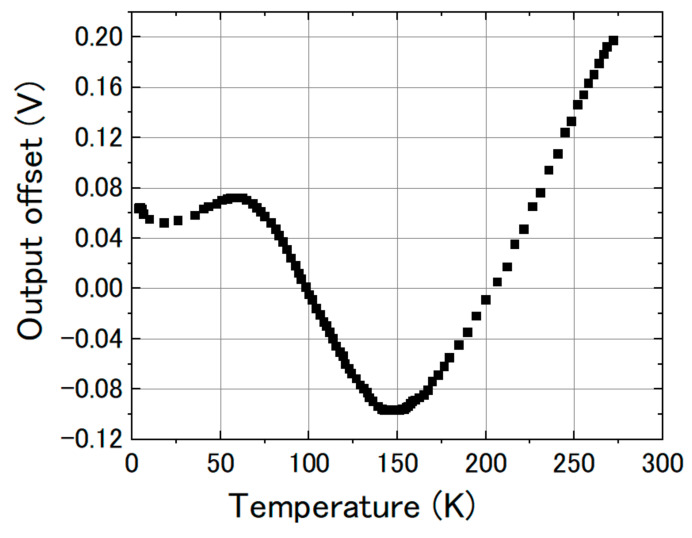
Output offset of the FeCoSiB magnetic sensors changing with the temperature.

From Figure 7, relative permeability of the FeCoSiB amorphous wire increased from 1406 to 1608, and the saturation flux density increased from 0.6 to 0.7 T when the temperature changed from room temperature to 77 K. Near the saturation, the change in the B–H curve at 77 K was not as steep as that at room temperature. This might have caused the deceasing of the signal amplitude at 77 K when the feedback was OFF (Figure 8). Only one cable was used to connect the sensing element of the magnetic sensor with the driving circuit. This design reduced the heat transferring though the cable and had the advantage of low-temperature operation of the magnetic sensor. From Figure 10, Figure 11 and Figure 12, the slope of the magnetic response changed from 0.4223 to 0.4092 and 0.4102 mV/mG when the operating temperature changed from room temperature to 77 and 4.2 K. The offset of the output signal changed from 271.482 mV to 62.823 mV/mG and 63.824 mV when the operating temperature changed from room temperature to 77 and 4.2 K. The offset change was not monotonic with temperature. We measured the output offset of the magnetic sensor changing the temperature. Figure 14 shows the results. There was a big change in the output voltage when the temperature changed from 4.2 K to room temperature: about 0.3 V in total (corresponding to about 0.75 Gauss). The output voltage decreased with the temperature to about 150 K, and then increased with the temperature. We cannot explain this phenomenon now—perhaps one reason is the shrinkage of the small plastic tube (the coil wound around it); another reason may be the abnormal thermal expansion of the FeCoSiB amorphous wire (abnormal thermal expansion often occurs for amorphous material). The DC bias current produces different magnetic fields at different temperatures. As a comparison, the offset change in the fluxgate was small, which was about 10 mG when the temperature changed from 4.2 K to room temperature. The temperature coefficient of the magnetic sensor limits its applications in some fields, such as operation in a wide temperature range, aerospace application or long-term magnetic field monitoring. We are developing bias reversal technology to reduce the temperature coefficient of this magnetic sensor.

## Figures and Tables

**Table 1:** Comparison of the fluxgate, anisotropic magneto resistance (AMR) and (Fe_0.06_Co_0.94_)_72.5_Si_2.5_B_15_ (FeCoSiB) amorphous wire magnetic sensors.

	Fluxgate	AMR Sensor	NIMS Sensor
Field resolution at 200 Hz	0.6 pT/√Hz	20 pT/√Hz	6 pT/√Hz
Field resolution at 2 Hz	1 pT/√Hz	200 pT/√Hz	20 pT/√Hz
Bandwidth	~1 kHz	1 MHz	10 kHz
Dimension	ϕ10 × 80 mm	12 × 10 × 2.5 mm^3^	ϕ2 × 5 mm
Number of connection wire	4 wires	8 wires	1 coaxial cable
